# Exploring the participation of young citizen scientists in scientific research: The case of iNaturalist

**DOI:** 10.1371/journal.pone.0245682

**Published:** 2021-01-19

**Authors:** Maria Aristeidou, Christothea Herodotou, Heidi L. Ballard, Alison N. Young, Annie E. Miller, Lila Higgins, Rebecca F. Johnson

**Affiliations:** 1 Institute of Educational Technology, The Open University, Milton Keynes, Buckinghamshire, United Kingdom; 2 Center for Community and Citizen Science, UC Davis School of Education, University of California Davis, Davis, California, United States of America; 3 California Academy of Sciences, San Francisco, California, United States of America; 4 Natural History Museum of Los Angeles County, Los Angeles, California, United States of America; Auburn University, UNITED STATES

## Abstract

Online citizen science projects have broadened options for accessing science and enabled different forms of participation in scientific research for adult and young volunteers. Yet, little is known regarding participation patterns among youth participants. Quantitative approaches were used to investigate the contribution of 183 young volunteers to citizen science on the iNaturalist platform and the participation behaviour that relates to their contribution. The participants accessed and used iNaturalist as part of one-day field-based events (bioblitzes) facilitated by museums. Compared to the observation behaviour of all iNaturalist users, as documented on the platform, the young volunteers observe fewer plants and birds, and more molluscs, arachnids and insects. The average daily contributions of young volunteers were found to be positively associated with a large proportion of active days on iNaturalist and a systematic contribution behaviour, yet negatively related to a long duration on the platform. This study enhances our understanding of young volunteers’ contributions to citizen science and provides insights for research on participation in online citizen science. Our findings have implications on how museums design the field-based events to encourage follow-up systematic participation and maintain active contribution.

## Introduction

Engaging people in scientific activities via Citizen Science (CS) has been discussed as an approach to opening up science to the public [1], advancing volunteers’ informal science education [2], empowering people with valuable skills for society [3], and contributing diverse perspectives to a more robust science [4]. Currently, there is a lack of agreement as to what CS is or means, which has resulted in considerable heterogeneity amongst CS projects, difficulties in assessing the quality of the projects and their outcomes, and a risk in establishing trust between citizens and scientists [5]. Towards this direction, the European Citizen Science Association (ECSA) [6] has produced a set of working principles that should underline good quality CS projects, which include involving volunteers in scientific activities that produce new knowledge, encouraging volunteers to take part in various stages of the scientific research and have access to the data and any publications, and providing feedback to volunteers about e.g., how their data are used, and are acknowledged by projects.

Understanding participation in online CS can inform the design of CS projects and increase success in terms of effectively engaging volunteers with scientific activities [7–10], and also enhance learning processes and outcomes [11, 12]. Such research can inform how more diverse audiences can be engaged with CS activities, given that most contributions are made by only a few volunteers, and that volunteers tend to only participate once or twice [10]. In particular, there is limited research on how young people participate and contribute in online CS programmes. Studies exploring young people's participation can inform the design of CS programmes that encourage young volunteers to contribute to CS initiatives and, in the long term, enhance their knowledge and interest in STEM topics and careers.

Several CS projects, while originally designed to contribute to scientific knowledge, attempt to achieve learning outcomes for volunteers [13]. Phillips et al. [14] have mapped these outcomes to six categories including personal interest to a CS project, self-efficacy to participate in the activity, motivation to achieve a science behaviour, knowledge of science content and the scientific process, development of science skills such as asking questions and designing studies, and behaviour, stewardship and civic action [14]. Amongst the benefits of participating in online CS is changing attitudes towards science, having a better understanding of the nature of science, increasing their science knowledge and additional topic-specific knowledge, and gaining generic knowledge about, for example, technology and language skills [3].

With regards to background characteristics, several studies note that CS volunteers are often white male, middle aged, educated, and with an interest in science [15, 16]. Their level and type of participation, especially in online CS projects, have been examined in several studies [7, 10, 17–23] that have clustered participants with common characteristics according to a set of metrics that captured online participation patterns. For example, the relationship between motivation and participation in the Old Weather project was explored via data analytics, surveys and interviews and categorised the participant contributions into high and low, with high contributors motivated by social or competitive features and low contributors relating to a more dabbling behaviour [7]. Volunteers on the Zooniverse projects of Galaxy Zoo and Milky Way were clustered via data analytics in profiles, representing their level of participation, such as hardworking, spasmodic, persistent, lasting and moderate [18]. Similarly, volunteers on nQuire-it were clustered into hardworking, persistent, loyal, lurking and visitor participation profiles, with extrinsic motivations (e.g., software) attracting and activating participants and intrinsic factors (e.g., interest in the topic) contributing to a longer stay in the project [10]. Finally, the participation of newcomers in Planet Hunters were analysed via data analytics and interviews and their activity was grouped into light work, intense viewing/contributing, careful annotation, talking & annotating, deep viewing & working and star specialisers [19].

In relation to the retention of participants in online CS projects, several studies (e.g., [20–25]) have reported that receiving feedback from the project is an important factor in the continuous participation of volunteers. For example, rapid reporting of the project results may have a positive impact on the participants’ retention [20], and providing feedback to participants with a science background, in person or even via email/other technology, can lead to continuous involvement in the project [23]. Similarly, Martin et al. [21] documented that scored-feedback by scientists in marine research projects was of great significance for the retention of stakeholders (e.g., divers and fishermen). Beyond feedback, other retention techniques include the use of online social networks for daily/weekly updates and notifications, and personalised messages to participants [22], while game elements either in the form of gamifying a mobile application or converting a CS activity into a game (See the case of Fold-it on Zooniverse) have been used to attract and motivate in particular young volunteers [24, 25].

One of the few studies focusing on young people examined the participation of 104 young volunteers on Zooniverse [12], showing that the majority of young people have irregular visits and long periods of inactivity. Another study suggested that, to engage young people with CS, simple interaction designs and a mobile-friendly implementation of CS projects are needed [26]. The use of CS in formal education showed that students’ skills such as data observation and correlations among many data, were confirmed or positively changed, and that participation in CS can be educationally beneficial in learning about aspects of the nature of science [17]. What has not yet been examined is the contribution behaviour of young people in online CS platforms, in particular iNaturalist (www.inaturalist.org), that rely on field observations made in events organised by museums (bioblitzes), and to which fields they contribute. Our study addresses this gap and goes a step further to investigate not only participation, but also the contributed organisms and how contribution relates to young volunteers’ participation behaviour.

The main aim of this research is to explore young iNaturalist volunteers, who participate in bioblitzes compared to the broader iNaturalist population who joined through other mechanisms, what they contribute to online CS, and what aspects of their participation relate to their contribution levels. iNaturalist is one of the largest online CS initiatives for naturalists, hosting approximately 50 million verifiable observations of 300,000 different species by 1,370,000 users as of this writing. This study is part of the LEARN CitSci research collaboration between three Natural History Museums (NHMs) and three research institutions in the UK and US, aiming to capture young people’s (aged 5 to 19 years old) participation and learning in online and field-based CS settings and to improve the design of CS programmes offered by NHMs. All three NHMs use and/or promote iNaturalist as the online CS platform focused on biodiversity. This is the first study that explores the level of participation and contribution on the iNaturalist platform and adds to the limited knowledge of how young people participate in online CS. Our findings have implications not only for online CS programs but also for how museums design the field-based events that use online platforms to encourage follow-up systematic participation online and maintain active contribution. The research questions (RQs) of this study are:

RQ1: *To what extent do young volunteers who participate in bioblitzes contribute on iNaturalist*, *and on what organisms do they predominantly focus*?
RQ2: *How does young volunteers’ participation behaviour (proportion of active days*, *duration*, *systematic participation) relate to their contribution (average daily contribution)*?

## Material and methods

### iNaturalist: Contributing to biodiversity research

iNaturalist (www.inaturalist.org) is a web-based and mobile-supported CS social network platform, where individuals can upload photo observations and identify organisms. Observations, which are photos of a single organism submitted by a user, are annotated by metadata such as date, time, location, whether the organism is captive/cultivated, taxonomic identification, and other user‐defined data fields. These can be particularly useful to scientists; for example, they can help in estimating species distribution (e.g., [27]), developing species checklists (eg., [28]), documenting introduced species (e.g., [29]), and describing new species (e.g., [30]). User profile pages are open to the public, and aggregated data are available for downloading by scientists, researchers, and other members of the public.

The taxon categories in iNaturalist are a communally curated synthesis of taxonomic ranks (kingdoms, phyla and classes). iNaturalist’s taxonomy (as presented on the platform) includes birds, protozoans, molluscs, insects, plants, reptiles, fungi, arachnids, ray-finned fishes, kelp, diatoms and allies, mammals, and amphibians. Examining the publicly available data on the website (11 May 2020), for example, we find the most observed organisms on iNaturalist are plants (41%), followed by insects (22%), and birds (16%). The least observed are protozoans with just 48,427 observations (0%). Unknown organisms (those currently unidentified) amount to 4% across iNaturalist. The overall observation of captive/cultivated organisms is 5% of the total [19, 31].

### Participants

The current study has been reviewed by, and received a favourable opinion, from The Open University Human Research Ethics Committee (reference number: HREC/3003/Herodotou), http://www.open.ac.uk/research/ethics/. The study explored the participation of 183 young people, 5–19 years old, who have participated in at least one bioblitz, on iNaturalist. Participants joined at least one Natural History Museum bioblitz (local field-based event) in one of the two partner museums that use iNaturalist as the data-recording platform, the Natural History Museum of Los Angeles (*n* = 56) and the California Academy of Sciences (*n* = 127). The bioblitzes took place between 2016–2019 and the age range of participants was acquired at their initial attendance in an NHM bioblitz by the event facilitators. Museums invited young volunteers to bioblitzes via social media (Facebook, Instagram and Twitter), on their websites, in newsletters, emails to previous attendees, and physical fliers/posters in the museum and museum events. In some occasions, partner organisations (e.g., San Francisco Zoo) would also advertise the bioblitz on their websites. Participants’ observations were publicly accessible and were retrieved from iNaturalist for the period between June 2013 and January 2020. In total, there were 37,209 observations recorded by this set of young people. The log files included information about each observation, such as usernames, date/time and location of the observation, taxonomic identification, and whether the organism was captive/cultivated. Prior to data analysis, the participant usernames were anonymised (e.g., iNat1, iNat2). The study received ethical clearance from the lead author’s university and the need for consent was waived by the ethics committee as iNaturalist contributions are publicly available.

### Contributions

Data analytics, visualisations, and correlations were used to analyse the observations made by young people. We investigated how young volunteers participate in iNaturalist by capturing their average daily contribution rate, the content of their submitted observations and characteristics of their participation. Descriptive statistics provided an overview of the young volunteers’ contributed organisms, insights into the average daily contribution, an understanding of whether the dataset was normally distributed, and a record of the super-users (outliers). Testing the normality of the data or lack of it facilitated our decision on using parametric or not parametric methods in the correlational analysis. The contributed organisms were represented by their iNaturalist taxon categories, as recorded in the log data files [32]. The average daily contribution was calculated as the ratio of total contributions per participant to the total days they remained contributing members of iNaturalist (from their first to their last contributing day) [33]. The average daily contribution was used as an approach to lessen the effect of participants’ different start dates.

### Participation metrics

Ponciano and Brasileiro [18] developed metrics to calculate the participation level of adult Zooniverse users in Milky Way and Galaxy Zoo projects. These metrics were then adopted by Aristeidou et al. [10] to explore the level of participation of adult Weather-it members on nQuire-it. Finally, these metrics were used by Herodotou et al. [12] to study young volunteers’ participation in Zooniverse. The latter is the only study exploring young people’s participation that would allow us to make comparisons between the findings of young people participating in different CS platforms.

**Fig 1:**
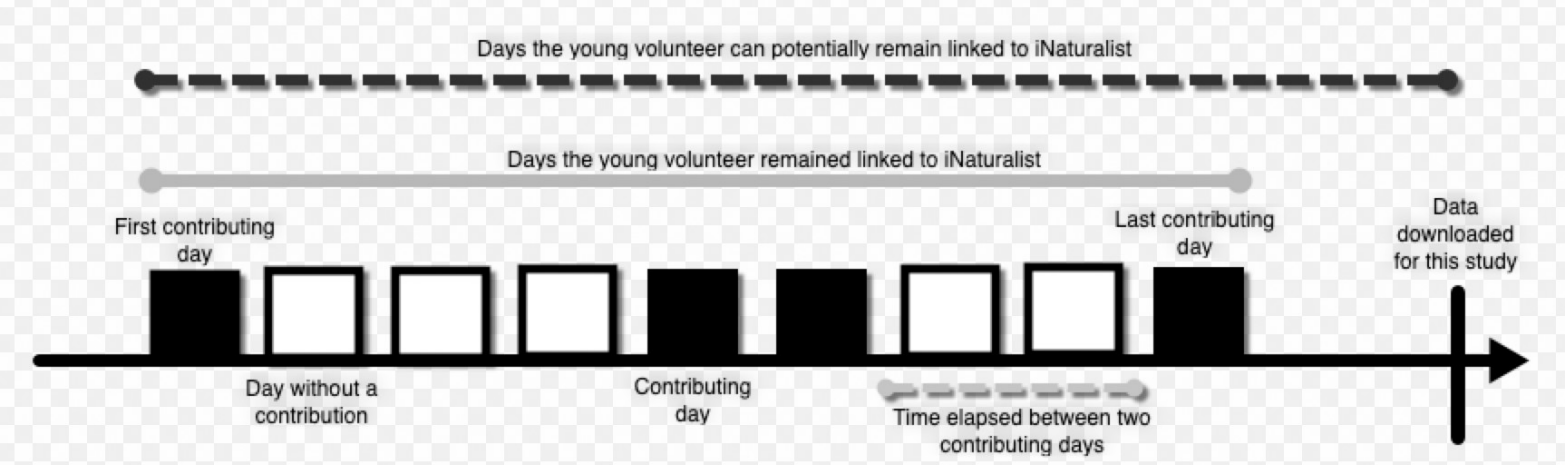
Timeline-example of a young volunteer on iNaturalist, with the contributing days and the first and last contributing day of their activity on iNaturalist. Figure inspired by Ponciano & Brasileiro [18]. The timeline (black arrow) starts with the first contributing day of a young volunteer and finishes on the date that data were downloaded for this study (31^st^ January 2020). The black boxes represent contributing days on iNaturalist and the white boxes represent days without a contribution. The small grey dotted line under the timeline counts the days between two consecutive contributing days. The grey line above the timeline indicates the days a young volunteer remained linked to the platform–from their first to their last contributing day. The black dotted line shows the days that a young volunteer could potentially be linked to iNaturalist–from their first day to the date that data were downloaded for this study (31^st^ January 2020).

The metrics were, therefore, also adopted in this study [21] (Fig 1). They are calculated as follows:

- *Activity ratio* is the ratio of days on which a user was active and contributed at least one observation in relation to the total days they remained linked to iNaturalist (from their first to their last contributing day). The closer to 1, the more active a user is during the days they are linked to the platform. This metric provides us with information on how **active in terms of contributing days** a young volunteer is on iNaturalist.
- *Relative activity duration* is the ratio of days during which a user is linked to iNaturalist (from their first to their last contributing day) to the total number of days they could have been linked. The latter is calculated from their first iNaturalist contribution day to the date that iNaturalist data were aggregated for this study (31st January 2020). The closer to 1, the longer a user remains on the platform. This metric provides us with information on the **duration** that a young volunteer is an active contributor on iNaturalist.
- *Variation in periodicity* is the standard deviation of the multiset of the number of days elapsed between each pair of sequential contributing days. The closer to 0 the steadier the rate by which a user returns to iNaturalist to contribute an observation. For instance, if a young participant submitted observations to iNaturalist on the 4rd, 6th, 10th, 22th and 27th August, the multiset that standard deviation would be applied to is {2, 4, 12, 5}. This metric provides us with information on how **systematically** a young volunteer contributes to iNaturalist.

All metrics values reported in this paper are means ± SD, unless otherwise noted.

An example of data in calculating metrics can be found in Table 1. The participants in the table below represent cases with large, moderate and small participation metric scores. User152 was active 451 out of the 1043 days that they were linked to iNaturalist (Activity ratio = 0.43) while User82 was active 3 out of the 360 days that they were linked to iNaturalist (Activity ratio = 0.01).

**Table 1:** Example of data—calculating metrics.

Participant	Active days	Total days linked to iNaturalist	First Day to 31^st^ January 2020	Activity Ratio	Relative Activity Duration	Variation in Periodicity (days)	Variation in Periodicity (normalised)
User152	451	1043	1056	.43	.99	2.07	0
User 25	168	362	524	.46	.69	2.42	0
User35	18	415	1056	.04	.39	72.76	.08
User82	3	360	1387	.01	.26	252.44	.29
User52	1	1	105	1.00	.01	N/A	N/A

The first four columns show the participant username, the number of active days, total days linked to iNaturalist, number of days between their first contributing day and the date that data were downloaded. The last four columns are calculations of the participation metrics (activity ratio, relative activity duration and variation in periodicity), based on the information given in the first columns. For example, User152 was active 451 out of the 1043 days that they were linked to iNaturalist (Activity ratio = 0.43).

### Correlations

SPSS (Version 25) was used for statistical analysis. First, the values of the metrics were normalised in the interval [0,1], when necessary (i.e. Variation in Periodicity). The degree of association between the participation metrics and the average daily contribution was assessed with the Spearman rank correlation, since none of the variables fulfilled the requirements for parametric testing. The effect size of significant findings was interpreted based on Cohen’s standard (Cohen, 1992). Correlation coefficients between .10 and .29 represent a small association, coefficients between .30 and .49 represent a medium association, and coefficients of .50 and above represent a large association or relationship, with a positive (+) sign indicating a positive relationship and a negative (-) sign indicating a negative relationship. The significance threshold was set at .05.

## Results

During the study period of six years and 7 months (time between first and last submitted observation), the 183 youth participants contributed 37,209 observations in total. Most of the observations took place in the United States, primarily in California where the two museums are located, but there were also international contributions (e.g. from Scandinavian countries, South America, and Southeast Asia, made while the users were traveling).

**Fig 2:**
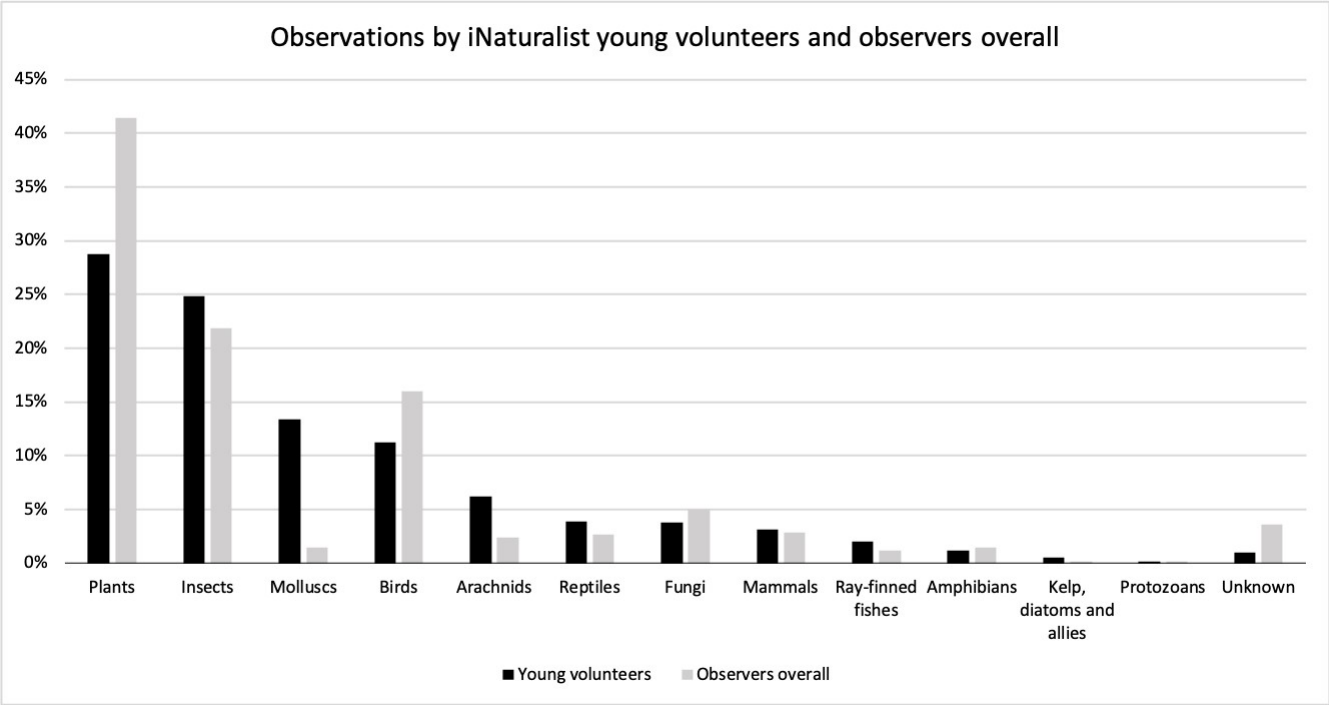
Comparison of contributed organisms by young volunteers and observers overall. This comparative bar chart shows the most observed organisms (%) by iNaturalist young volunteers taking part in this study (black bars) against the most observed organisms (%) observed by all iNaturalist users (grey bars). Young volunteers observe fewer plants and birds, but more molluscs, arachnids and insects.

The most observed organisms by young people, in descending order, were plants (29%), insects (25%), and molluscs (13%). Protozoans were the least observed organism with just 41 contributions (<1%). A number of contributed observations (325; 1%) remain unknown. Captive/cultivated organisms made up 4% of young volunteers’ contributions (1,649 observations), which is lower than the 5% of iNaturalist observers overall. Compared to iNaturalist observers overall (Fig 2) young volunteers observe fewer plants, with a percentage difference (PD) of 12%, and slightly fewer birds (PD = 5%). They, however, observe more molluscs (PD = 12%) and slightly more arachnids (PD = 4%) and insects (PD = 3%).

**Fig 3:**
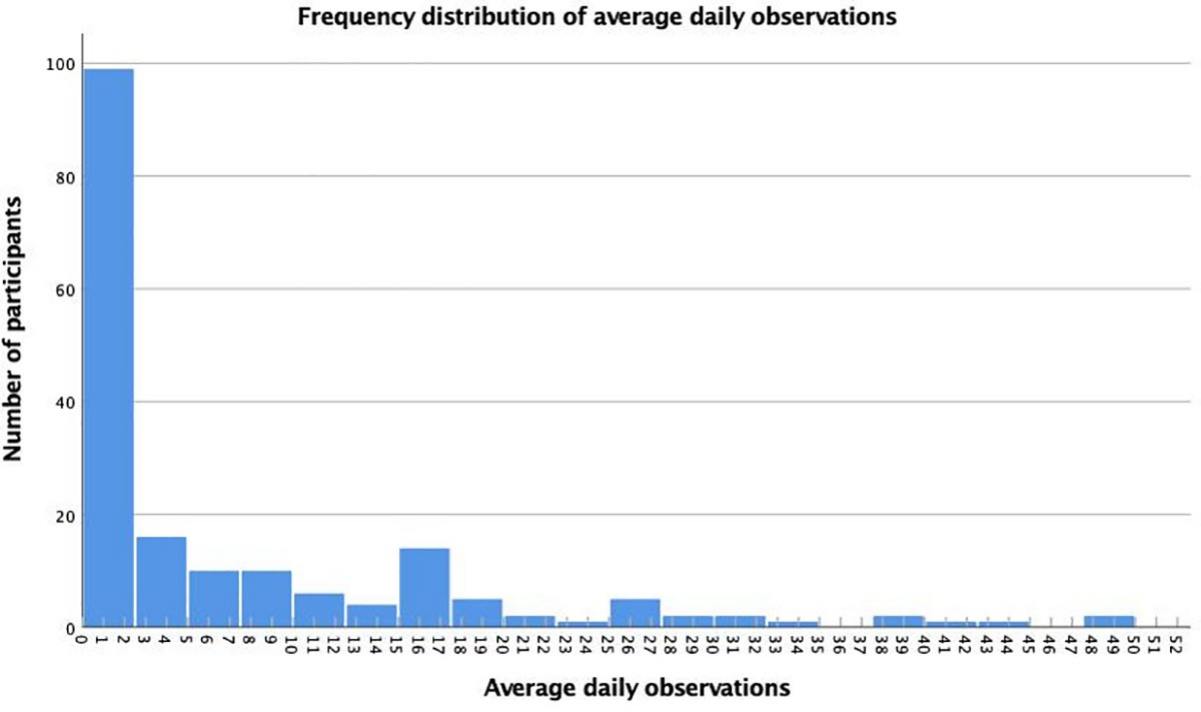
Frequency distribution of observations by young people. The histogram presents how many average observations young volunteers contributed daily on iNaturalist. The right skewed shape of the graph shows the asymmetrical daily contributing pattern with a large number of participants (n = 81) contributing less than 1 observation daily while 12 participants contribute beyond average (>27 observations).

The median number of average daily contributions per volunteer was 1.99 ± 10.292, with participants contributing from 0 to 48 observations on average each day they were linked to iNaturalist. Fig 3 represents the distribution of the average daily number of observations by young volunteers. The shape of the graph is skewed right, highlighting an asymmetrical daily contributing pattern, with a large number of participants (n = 81) contributing less than 1 observation daily. Twelve participants (7%) made contributions beyond the upper boundary of average daily observation numbers (27 observations); these participants were considered as outliers and coined as super-users. Even within this group, there was a large difference between the first super-user (48 daily observations) and the second super-user (27 daily observations).

The results of the three participation metrics (activity, duration and systematic contribution) of participants on iNaturalist were as follows: Activity ratio (activity): 0.53 ± 0.45; Relative Activity Duration (duration): 0.26 ± 0.38; Variation in Periodicity (systematic contribution): 79.68 ± 128 (0.09 ± 0.15). Variation in Periodicity was calculated with n = 75, since a large number of participants had one or two contributing days only on iNaturalist. Young people, on average, were found to be actively contributing during 53% of the days they were linked to iNaturalist (Activity ratio), they remained active contributors on iNaturalist for the 26% of the total time that they could be active (Relative Activity Duration), and they did not contribute to the platform systematically, with an average variation of 80 days between their contributing days (Variation in Periodicity). When calculating the active (contributing) days that participants had on iNaturalist, we note that the majority of participants (59%) had one or two contributing days only.

Table 2 shows the descriptive statistics of participation metrics in this study in comparison to those of Milky Way and Galaxy Zoo [18], nQuire-it [10] and Zooniverse (all projects) [12].

**Table 2:** Comparison of average participation metrics in five online CS projects and platforms.

	Activity Ratio	Relative Activity Duration	Variation in Periodicity (days)
**Adult volunteers**			
Galaxy Zoo [18]	0.33±0.38	0.23±0.29	25.23±49.16
Milky Way [18]	0.40±0.40	0.20±0.30	18.27±43.31
nQuire-it [10]	0.32±0.35	0.43±0.44	5.11±5.36
**Young volunteers**			
Zooniverse (all projects) [12]	0.31±0.44	0.49±0.36	49.93±52.08
**iNaturalist**	**0.53±0.45**	**0.26±0.38**	**79.68±128**

The results of three participation metrics across five different online citizen science projects and platforms, three researching adult participation behaviour and two researching young volunteer behaviour. Metrics values are means ± SD.

Correlations were computed among the three participation metrics and the average daily contribution metric on data for the 183 young iNaturalist volunteers (Table 3). The Spearman correlation suggests that all the six correlations were statistically significant. The Average Daily Contribution has a strong significant positive association with Activity ratio (rs(183) = .896, p < .001) and significant strong negative associations with Variation in Periodicity (rs(75) = - .824, p < .001) and Relative Activity Duration (rs(184) = - .710, p < .001). The Activity ratio has significant strong negative associations with Variation in Periodicity (rs(183) = -.924, p < .001) and Relative Activity Duration (rs(183) = -.819, p < .001). Finally, the Variation in periodicity has a significant weak positive association with Relative Activity Duration (rs(183) = .283, p = .014).

**Table 3:** Correlation matrix between the participation metrics and the average daily contribution.

Metrics	1	2	3	4
1. Activity ratio	1			
2. Relative Activity Duration	-.819*	1		
3. Variation in Periodicity ^a^	-.924*	.283*	1	
4. Average daily contribution	.896*	-.710*	-.824*	1

The Spearman correlation coefficients between each set of participation metrics (activity ration, relative activity duration and variation in periodicity) and the average daily contribution. The Average Daily Contribution has a strong significant positive association with Activity ratio (rs(183) = .896, p < .001) and significant strong negative associations with Variation in Periodicity (rs(75) = - .824, p < .001) and Relative Activity Duration (rs(184) = - .710, p < .001). The significance threshold is set at .05.

^a^ n = 75

*Correlation is significant at the 0.05 level (two-tailed)

## Discussion

### Level and type of participation

RQ1: To what extent do young volunteers who participate in bioblitzes contribute on iNaturalist, and on what organisms do they predominantly focus?

The most contributed organisms by iNaturalist young volunteers were plants, insects, and molluscs. A comparison between the total contributed organisms by iNaturalist observers overall [19] to the ones coming from young volunteers who participate in bioblitzes demonstrates some common patterns (such as plants and insects ranking first in both groups’ contributions), which reflects the relative ease of observing these categories of organisms. Nevertheless, it highlights some dissimilarities, including an altered relative proportion of contributed organisms: young volunteers, who participate in bioblitzes, tend to observe fewer plants and birds and more molluscs, arachnids and insects. This different relative importance may have several explanations: an increased (e.g., insects, molluscs) and decreased (e.g., birds, plants) preference of young volunteers towards some organisms; young volunteer’s location (i.e., California) and local environment; participant attendance at a bioblitz event or joining a museum-run taxon-specific project. Another explanation about the lower proportion of bird observations might be the lack of the right equipment by young people, for example, digital single-lens reflex (DSLR) cameras, that would allow them to capture their observation in a verifiable picture. The proportion of young volunteers’ contributions of captive/cultivated observations was slightly smaller than that of the overall contributions, while that of unknown organisms was four times smaller. The latter finding may be due to the fact that all of the young participants came from bioblitzes, and were probably supported in making identifications in iNaturalist, either through being taught how to use the machine learning mechanisms or by a facilitator.

Our findings indicate that young people, who have contributed in at least one bioblitz, usually contribute an average of 0 to 26 daily observations, with a small proportion of participants labelled as super-users who have an extremely high average daily number of contributions. Moreover, the majority of participants had one or two contributing days only. An explanation for the high number of these young volunteers may be that most of the participants accessed and used iNaturalist as part of a one-day field-based bioblitz event facilitated by museums, and had no facilitation or support thereafter. These findings raise questions as to whether young people perceive iNaturalist observations as something to do in nature or with professionals, how the design of field-based events (e.g., bioblitzes) could be modified to encourage follow-up participation on iNaturalist, as well as what actions could be taken thereafter to maintain young participants on iNaturalist. One way would be for bioblitzes to highlight the fact that young participants can use iNaturalist no matter the location, including their own yards. iNaturalist projects aiming at documenting the biodiversity in our residential landscapes (for example, Yardversity), could further promote follow-up participation.

This asymmetrical participation aligns with previous research on adults [10, 18] and young people [12] in CS activities, highlighting that a few volunteers make the majority of contributions in CS, while the majority of them contribute only once or twice. This finding has different implications for the scientific research goals of the CS project, whereby recruiting and fostering those super-users might be the best strategy, as compared to the outreach goals of a project, whereby engaging a large number of people over time is a success.

Particularly in light of the only other published research on CS participation by young people, we see some similarities and differences between our findings and Herodotou et al.’s [12] findings with the Zooniverse youth volunteers (see Table 2). iNaturalist youth volunteers were contributing during a larger proportion of days, but for a shorter period of time and with less systematic contribution. Compared to adult levels of participation in Zooniverse Galaxy Zoo project, Zooniverse Milky way project [18] and nQuire-it [10], we found that overall young volunteers on both platforms, and mainly iNaturalist, have less systematic contribution. However, iNaturalist young volunteers had a larger proportion of active days than Zooniverse young volunteers, with the latter approaching the lower activity levels of adults. These variations may be explained by distinct design features in each platform [10]. For instance, the higher activity but lower systematic participation in iNaturalist, compared to Zooniverse, may be due to the main use of the iNaturalist platform, which for the majority of the youth we studied, was to collect data in NHM-led field-based bioblitz events. The findings could also be explained by the mode of the two platforms, with Zooniverse functioning exclusively online while iNaturalist requires time spent outdoors, and young people’s preference or availability when engaging with citizen science activities.

In contradiction with other research that focuses solely on the number of contributions (e.g., [7]), this study looked as well at the activity, duration, and systematic contributions. As a whole, iNaturalist young volunteers contribute half of the days they are linked to iNaturalist, they remain linked to iNaturalist nearly one out of the four days they could potentially be linked, and they contribute every 80 days on an average.

### Relationship between participation level and contribution

RQ2: How does young volunteers’ participation behaviour (proportion of active days, duration, systematic participation) relate to their contribution (average daily contribution)?

The participation metrics facilitated our understanding of young people’s participation behaviour and how this behaviour associates with their average daily contributions. The Spearman’s rho revealed that young volunteers with a higher proportion of active days (Activity ratio), more systematic contribution (Variation in Periodicity), and fewer days linked to iNaturalist (Relative Activity Duration) tend to have a higher average daily contribution. This finding suggests that encouraging young volunteers to contribute more systematically and have more active days is more likely to increase their number of contributions and therefore, to promote a more active participation. This finding is accompanied by a strong negative relationship between young volunteers’ duration on iNaturalist and their activity and systematic visits. Both findings have implications for citizen science and NHM programs that aim to use bioblitzes and other one-time events to recruit young people into sustained science engagement; if young people who participate in bioblitzes never return to iNaturalist, then these events do not equal long term participation in science by youth. The observed drop outs could be attributed to the lack of retention efforts that promote the continuous participation of bioblitz visitors on the platform, or lack of access to nature and the lack of captive organisms at home. Research into solving the retention issue is already underway as part of the LEARN CitSci project, with NHM facilitators employing feedback, suggested by previous work with adults [21–23, 34], or potentially introducing game elements as in other citizen science projects [24, 25] as an approach to maintain young people’s interest in the platform. Future studies should also compare how particular technological affordances and distinct design characteristics in online CS can activate different types of participation behaviour or may become a barrier or enabler of high levels of activity and systematic contribution for young volunteers.

In the future, researchers could explore how museum strategies and platform approaches for reaching and retaining young people may result in different patterns of participation. This prospect of being able to understand and improve how young volunteers contribute to iNaturalist, serves also as an incentive for looking into the participation of adult iNaturalist volunteers. Findings of a comparison between adult and young people’s participation on iNaturalist would strengthen our understanding of the particular factors that may affect young people’s preferences and participation.

### Limitations

A limitation encountered while replicating the method developed by Ponciano and Brasileiro [18] was the absence of data for calculating daily devoted time. This metric would be useful for recording the actual time young people engage with iNaturalist activities, for example, browsing and viewing other people’s observations. Exploring young people’s identification behaviour would have been a good way to mitigate this limitation; however, as there is no way for automated extraction of identification on iNaturalist, there is a high risk of error due to oversight. Similarly, manual collection of demographics could lead to high error because not all of the iNaturalist users disclose such personal information in their public profiles. Moreover, this study has potential sample limitations. Young volunteers of this study may not be representative of young people who participate in bioblitzes in other locations, or young people who have never attended a bioblitz. Our decision to focus on young volunteers participating in the particular museum bioblitzes is based upon selection limitations, since the iNaturalist platform does not disclose personal information of participants, such as their age or location, and there is no way to identify and automatically extract data for the particular group of participants. Also, the sample size is relatively small (<200) hence any outcomes should be discussed within the context of this study and interpreted with caution, rather than being generalised to the population. Future research could focus on ways of identifying iNaturalist young people via other channels and exploring the characteristics and participation behaviour of young volunteers overall.

### Implications for research and bioblitz design

This study is the first step towards enhancing our understanding of what young volunteers on the iNaturalist platform contribute to online CS and what relates to their contribution levels. Taken together, our findings highlight the importance of designing field-based events that promote follow-up participation in CS online, and the significance of designing online CS platforms that embody retention mechanisms and techniques. Overall, our research reveals the active contribution of young people, who attended bioblitzes, in online CS. Further, it underlines the importance of treating the participation behaviour and retention of young people, who participate in bioblitzes, differently than those of other participant groups. Compared to adult volunteers, young volunteers may be characterised by different capabilities, availability of instruments, outdoor opportunities and personal preferences. Also compared to young people who have never participated in a bioblitz, they may be characterised by different motivations, participation frequency and opportunities, and contribution content and rate. Our findings add to a growing body of literature on participation in online CS and constitute an excellent initial step towards understanding and supporting the contribution of young people in this growing field.
